# Genetic basis and network underlying synergistic roots and shoots biomass accumulation revealed by genome-wide association studies in rice

**DOI:** 10.1038/s41598-021-93170-3

**Published:** 2021-07-02

**Authors:** Yan Zhao, Zhigang Yin, Xueqiang Wang, Conghui Jiang, Muhammad Mahran Aslam, Fenghua Gao, Yinghua Pan, Jianyin Xie, Xiaoyang Zhu, Luhao Dong, Yanhe Liu, Hongliang Zhang, Jinjie Li, Zichao Li

**Affiliations:** 1 State Key Laboratory of Agrobiotechnology/Beijing Key Laboratory of Crop Genetic Improvement, and College of Agronomy and Biotechnology , China Agricultural University, Beijing, 100193 People’s Republic of China; 2 State Key Laboratory of Crop Biology, Shandong Key Laboratory of Crop Biology, College of Agronomy, Shandong Agricultural University, Tai’an, 271018 Shandong People’s Republic of China; 3 Guangxi Key Laboratory of Rice Genetics and Breeding, Rice Research Institute of Guangxi Academy of Agricultural Sciences, Nanning, 530007 Guangxi People’s Republic of China

**Keywords:** Computational biology and bioinformatics, Genetics, Plant sciences

## Abstract

Genetic basis and network studies underlying synergistic biomass accumulation of roots and shoots (SBA) are conducive for rational design of high-biomass rice breeding. In this study, association signals for root weight, shoot weight, and the ratio of root-to-shoot mass (R/S) were identified using 666 rice accessions by genome-wide association study, together with their sub-traits, root length, root thickness and shoot length. Most association signals for root weight and shoot weight did not show association with their sub-traits. Based on the results, we proposed a top-to-bottom model for SBA, i.e. root weight, shoot weight and R/S were determined by their highest priority in contributing to biomass in the regulatory pathway, followed by a lower priority pathway for their sub-traits. Owing to 37 enriched clusters with more than two association signals identified, the relationship among the six traits could be also involved in linkage and pleiotropy. Furthermore, a discrimination of pleiotropy and LD at sequencing level using the known gene *OsPTR9* for root weight, R/S and root length was provided. The results of given moderate correlation between traits and their corresponding sub-traits, and moderate additive effects between a trait and the accumulation of excellent alleles corresponding to its sub-traits supported a bottom-to-top regulation model for SBA. This model depicted each lowest-order trait (root length, root thickness and shoot length) was determined by its own regulation loci, and competition among different traits, as well as the pleiotropy and LD. All above ensure the coordinated development of each trait and the accumulation of the total biomass, although the predominant genetic basis of SBA is still indistinguishable. The presentation of the above two models and evidence of this study shed light on dissecting the genetic architecture of SBA.

## Introduction

Asian cultivated rice (*Oryza sativa* L.) is the staple food of half the world’s population^1^. Faster biomass accumulation (shoot and root biomass) is a guarantee of high yield of rice while maintaining the current harvest index (HI)^1–3^. In the past, conventional rice breeding mainly focused on the selection of some agronomic traits in the aboveground part^4^. It has been challenging breeding for strong roots and phenotype-based selection of ideal proportion of aboveground and underground parts^5, 6^. Generally, conventional breeding approaches are usually phenotype-oriented and selections are ineffective for improvement of root traits^7^. Recently, rational design breeding based on the extensively accumulated knowledge about the genes that regulate important agronomic traits has been successfully implemented in pyramiding multiple complex agronomic traits^4^. In light of the previous knowledge-based, insights into the genetic architecture underlying synergistic roots and shoots biomass accumulation (SBA) could provide valuable information for high-biomass rice breeding.

Root weight, shoot weight and the ratio of root-to-shoot mass (R/S) are three main traits directly related to biomass accumulation of plants, and they are further determined by their sub-traits, such as root length, root thickness, shoot length etc. Recently, a large number of quantitative trait loci (QTLs)/genes were identified for the six traits/sub-traits above using mutant and bi-parental populations, and most of them had been verified in transgenic plants or introgression lines^8–15^. However, it is still hard to pinpoint effect of these genes in rice with different genetic backgrounds, for its rich-within-species diversity with two major subspecies, *indica* and *japonica* and differentiation of subpopulations^16^. Fortunately, the most commonly used morphological characters related to biomass had been reconstructed in selecting and crossing procedures during rice domestication^17, 18^. Genome-wide association studies (GWAS) enabled identification of numerous SNPs associated with targeted traits in diverse rice accessions, which could be more valuable with stable- and high-effect under diverse genomic background. Lately, a great number of loci for rice biomass traits have been detected by GWAS, which confirmed that variation exists both within and between rice subpopulations for these traits^19–21^. However, in-depth knowledge of the natural variation in genes underlying SBA is required with respect to identifying or designing superior cultivars with greater biomass, especially root biomass.

Furthermore, dissection of a sub-trait is insufficient for molecular breeding for complex quantitative traits constrained by their multiple sub-traits because many traits correlate with one another and tend to be tightly integrated, resulting in heritable co-variation^22, 23^. As a matter of fact, correlations amongst traits are common phenomenon in biology, which have been detected among traits in determination of panicle^24^, growth duration and yield^25^ and root and shoot traits in rice^26^. Breeders need to overcome disadvantageous correlations to meet production requirements while to improve target trait(s). Integrated intracellular and intercellular signaling networks through roots and shoots enable trait correlations and have been confirmed in previous studies^27–29^, but their role and genetic mechanism in SBA remain elucidated. Additionally, pleiotropy and linkage disequilibrium (LD) in natural population are usually considered as the main reasons for genetic trait correlations, although the predominant genetic basis of trait correlations is controversial^24, 30^. Previous study discriminates between genic and true pleiotropy^30^. Genic pleiotropy refers to a gene affecting two or more traits simultaneously. Because in this case genes represent the smallest genetic unit, genic pleiotropy can be caused by intragenic linkage of QTNs, each affecting different traits, or true pleiotropy. True pleiotropy is due to a QTN affecting two or more traits. In the modern era, a lot of pleiotropy gene and pleiotropy genome regions have been detected in rice, such as *Ghd7*^31^, *OsSPL16*^32^ and *qRT9*^12^, but the research on discrimination of LD from pleiotropy is insufficient. Therefore, understanding of how roots and shoots coordinate development at molecular level is essential for the genetic improvement of roots and shoots biomass accumulation.

In this study, we measured root weight, shoot weight, R/S and their sub-traits including root length, root thickness and shoot weight, followed a correlational analysis among the six traits related to biomass accumulation. To explore new or subpopulation-specific loci underlying SBA, GWAS were performed in whole association panel and its two subpopulations respectively. Most of the loci can be validated by comparison of the GWAS results and known QTLs/genes. Subsequently, we explored interactions among the loci and the genetic network across SBA based on these loci. Meanwhile, an intragenic linkage event of quantitative trait polymorphic-nucleotides (QTNs) was detected within a pleiotropic gene for root weight and root length, while each QTN was affecting a different trait. The results shed light on how roots and shoots develop synergistically as well as information that are useful for future molecular applications of these loci in breeding for high biomass.

## Materials and methods

### Materials and sequencing

A total of 665 Asian cultivated rice accessions and one African cultivated rice accession (*Oryza glaberrima* L.) was used in the present study, which obtained from the 3000 Rice Genome Project (3KRGP)^16, 19, 33^. The set included 239 accessions from a mini core collection of Asian cultivated rice in China^34^ and 427 accessions in the International Rice Molecular Breeding Network^35^. Genome sequencing data (12,627,485 SNPs) of 666 rice accessions were obtained from the 3KRGP^16^. Imputation was performed to infer missing genotypes using Beagle 4.0 software^36^. After removing SNPs with minor allele frequency (MAF) ≥ 0.05 and heterozygote rates ≤ 50%, a common SNP set was constructed for further analysis using an in-house Python script.

### Phenotyping of six traits for root and shoot biomass accumulation

A hydroponic culture experiment comprising 666 rice accessions (five seeds of each accessions) was conducted twice as two replications at China Agricultural University in 2014 and obtained accurate reproducible phenotypic data of root and shoot biomass. Method for the experiment and determination method of six seedling traits were described in Zhao et al.^19^. In short, seed placed for germination for 64 h after washing with distilled-water. Five uniformly germinated seeds of each accession were placed into five wells on a plastic foam frame. Two frames were floated in a plastic box containing Yoshida nutrient solution^37^, and the pH was adjusted to 5.5. Plants were grown under natural conditions for 23 days and then five plants per accession were sampled and measured for root weight, shoot weight, shoot length, root length and root thickness. The R/S was computed to evaluate the coordination between growth and development of the roots and shoots. The mean values of all five plants of each accession represented the phenotypic data of the accession. The collection and phenotyping of the 666 rice accessions comply with international guidelines and legislation.

### Genome-wide association study

It is usually confounding for population structure evaluation because of the non-independence of SNPs caused by strong LD. When independent SNP numbers were determined by PLINK (window size 50, step size 50, *R*^2^ ≥ 0.3)^38^, the population structure, principle components (PC) and phylogenetic relationship of the associations were determined by Admixture, GAPIT and Tassel software^39–42^.

GWAS on full population of *indica* and *japonica* were performed using GAPIT under the FarmCPU model^43^. Here, the top three PCs were used to estimate population structure. Independent SNP numbers in full population (295,881), *indica* (307,023) and *japonica* (130,088) were calculated for an appropriate threshold, given that it is too stringent for significant association detection when the threshold is derived from the total number of markers^44, 45^. The suggested thresholds (full population: 3.38 × 10^−6^, *indica*: 3.26 × 10^−6^ and *japonica*: 7.69 × 10^−6^) of associations were further calculated by Genetic Type I error calculator (GEC) software to control the genome-wide type I error rate at 0.05 by using Bonferroni-adjusted correction^44^. Finally, the threshold was defined at − log(*p*) = 6 for full population, *indica* and *japonica* together.

### Association network

We constructed association network using Cytoscape 3.5.1^46^, in which six traits and their association panel were represented by nodes, while the edge represented the LD between signals and link, and also traits and signals. The nodes for six traits were uniform, and the sizes of other nodes for signals were determined by –log(*p*) value. Meanwhile, edges between traits and signals were uniform, and edges between each two signals represented their LD value. Pairwise *R*^2^ values were calculated between all significant SNPs using PLINK^38^.

## Results

### Population structure and variations in plant biomass of 666 cultivated rice accessions

To investigate the variation in the plant biomass and R/S in rice, a population of 666 cultivated rice accessions was constructed, including 665 Asian cultivated rice accessions and one African cultivated rice accession (Table S1). The population was collected from 41 countries worldwide, which represented large variations in the geographical origin and diversity of genotype and phenotype reported in previous studies^16, 19^. The sequencing data of the 666 accessions were directly retrieved from 3KRGP^16^. A total of 4,504,569 SNPs were identified after imputation and removing the SNPs with minor allele frequencies (MAF) < 0.05 and heterozygote rates > 50% (Methods), and used for further GWAS. The population structure analysis showed that *indica* and *japonica* varietal groups appeared clearly at *K* = 2 based on the 295,881 independent SNPs (Methods), which was supported by neighbor-joining tree (Fig. S1). PC analysis confirmed that the top three PCs explained 0.23, 0.03 and 0.03 of the genetic variation within the population (Fig. S1). Hence, we classified the population into two major subspecies, *indica* (414) and *japonica* (252) for further phenotypic analysis and GWAS on two subpopulations, though there were several typical *indica* and *japonica* at the junction between the clouds with PC plots and the phylogenetic tree (Fig. S1 and Table S1).

**Figure 1:**
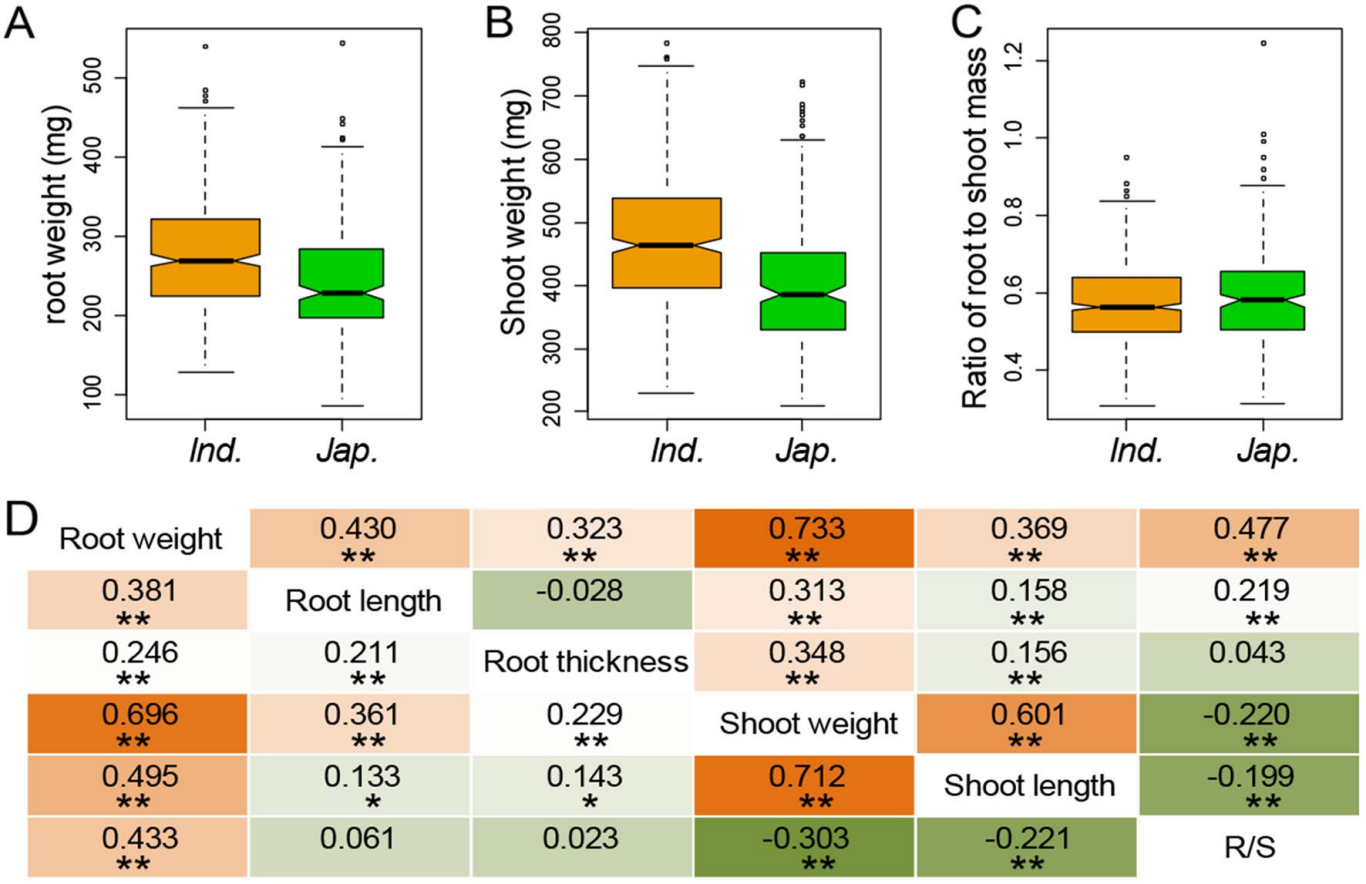
Subpopulation characteristics and trait correlations of six biomass traits. Phenotypic distributions of (**A**) root weight, (**B**) shoot weight and (**C**) ratio of root-to-shoot mass (R/S), divided by the *indica* and *japonica*. (**D**) A heatmap depicting Pearson’s correlation coefficients between phenotype means in *indica* (lower triangle) and *japonica* (upper triangle) for six traits.

Root weight, shoot weight and R/S of 666 rice accessions were investigated using hydroponics for two sub-traits of root weight (root length and root thickness) and one shoot weight sub-trait (shoot length) (method). The rice population showed significant numbers of variations for root weight (27.2%), shoot weight (24.8%) and R/S (11.4%), along with their sub-traits including root length (12.5%), root thickness (9.2%) and shoot length (15.2%) (Fig. S2 and Table S1). Comparison of biomass between the two main rice subspecies indicated that the average root weight and shoot weight of *indica* (276.5 and 469.4 mg) were higher than those of *japonica* (242.8 and 401.1 mg) with extreme significance at *p* value < 1.0 × 10^−8^ by Independent-sample T-test (Figs. 1, S3 and Table S2). However, *indica* R/S was lower than *japonica* with accordance significance value (*p* < 0.05). Among these sub-traits, signal strength for root thickness of *japonica* was significantly greater than those in *indica*, while no obvious difference was detected in root length and shoot length between the subgroups (Fig. S3 and Table S2). These results confirmed that *indica* is more capable of accumulating plant biomass than *japonica*, while *japonica* has a tendency to accumulate more root biomass. The higher correlation detected between shoot weight and its sub-trait shoot length with Pearson correlation coefficient at 0.601, 0.712 and 0.632 in *indica*, *japonica* and the full population respectively. On the contrary, the correlations were lower between root weight and its sub-traits including root length and root thickness (Figs. 1D, S4). Interestingly, the highest correlation was detected between root weight and shoot weight in the whole population, *indica* and *japonica* with Pearson correlation efficiencies of 0.737, 0.733 and 0.696, respectively (Figs. 1D, S4). The results suggested that there were superior genes or networks influenced the collaborative development of roots and shoots, by regulating subordinate genes or networks associated with root length, root thickness and shoot length.

### Identification of associated loci for six seedling traits

**Figure 2:**
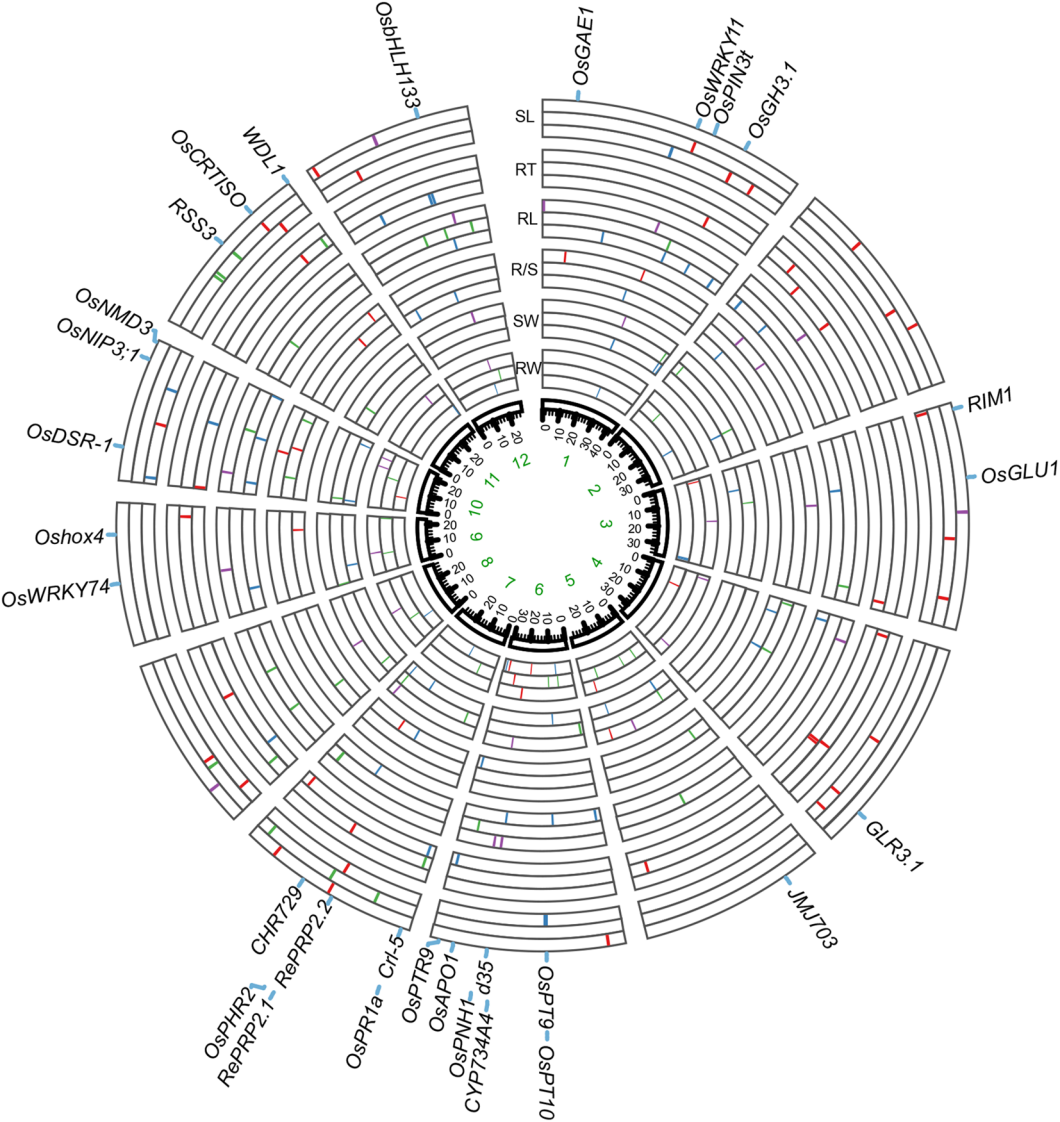
Circos map of all association signals for six traits of rice seedling biomass in the full population (inner layer), *indica* (middle layer) and *japonica* (outer layer). Signals identified in full population, *indica* and japonica are colored in blue, green and violet, and signals colored red show QTLs have observed in previous studies. Thirty known genes around the association signals are labeled in the outermost layer.

GWAS for six seedling traits were performed in the full population with first three PCs and trait-special kinship (Method) using FarmCPU model^43^. As QQ plot showed that FarmCPU controlled efficiently population structure and relationships because there was no inflated *p* values and the majority (99%) of markers exhibited *p* values equal to or lower than the expected with accordance null hypothesis (Fig. S5). Thus, the GWAS results were presented under Farm CPU and a threshold of − log_10_(*p*) = 6 was determined after Bonferroni-adjusted correction (Method). We identified 14, 13, 10, 17, 15 and 20 significant SNPs (significant signals) for root weight, shoot weight, R/S, shoot length, root length and root thickness respectively in the full population (Figs. 2, S6 and Table S3). Here we defined that each association signal represented a 500 kb region around the significant SNPs, given LD decay values of ~ 123 kb and ~ 167 kb in *indica* and *japonica* populations respectively^17^. All candidate genes in the region could be related to corresponding trait.

Population structure was analyzed. Phenotypic differentiation between both subpopulations have been detected in the association panel and suggested that there were subpopulation-specific causal SNPs for rice seedling biomass. Then we performed GWAS within two subpopulations to explore subpopulation-special association signal for six traits related to biomass using Farm CPU in *indica* and *japonica*, respectively (Figs. S7, S8). Finally, we detected 14, 13, 8, 13, 11, 13 and 11, 8, 7, 9, 0, 9 association signals in *japonica* and *indica* for 6 traits, respectively (Figs. 2, S9, S10 and Table S3). As predicted, no common association signal was detected between *indica* and *japonica* for each of any six traits in rice. The results indicated that a substantial portion of the genetic variation responsible for seedling biomass remains isolated within individual subpopulations of rice.

The analysis of phenotypic variation explained (PVE) indicated that the most of associations accounted for low phenotypic variation with average PVE at 2.99%, 3.98%, 3.10%, 4.68%, 3.01% and 3.46% for root weight, shoot weight, R/S, shoot length, root length and root thinkness, respectively (Table S3). To validate these associations for the six seedling traits, we compared the associations with QTLs detected by linkage mapping in previous studies. There were 8/29, 24/26, 23/42 and 8/25 association signals within known QTLs for root weight, root length, root thickness and R/S, respectively^10^, while few linkage mappings focused on shoot length and shoot weight (Fig. 2 and Table S3). Furthermore, many genes with known function in shoot development were detected around association signals of corresponding traits as well as that in root development, with six genes for root weight, four genes for root length, eight genes for root thickness, six genes for R/S, three genes for shoot weight and eleven genes for shoot length. Detailed information of each gene for the 6 traits was provided in Table S3. The large proportion overlapping between association signals and results from previous studies suggested that these loci were responsible for natural variation of the corresponding traits.

### Genetic basis of variation in rice biomass accumulation

**Figure 3:**
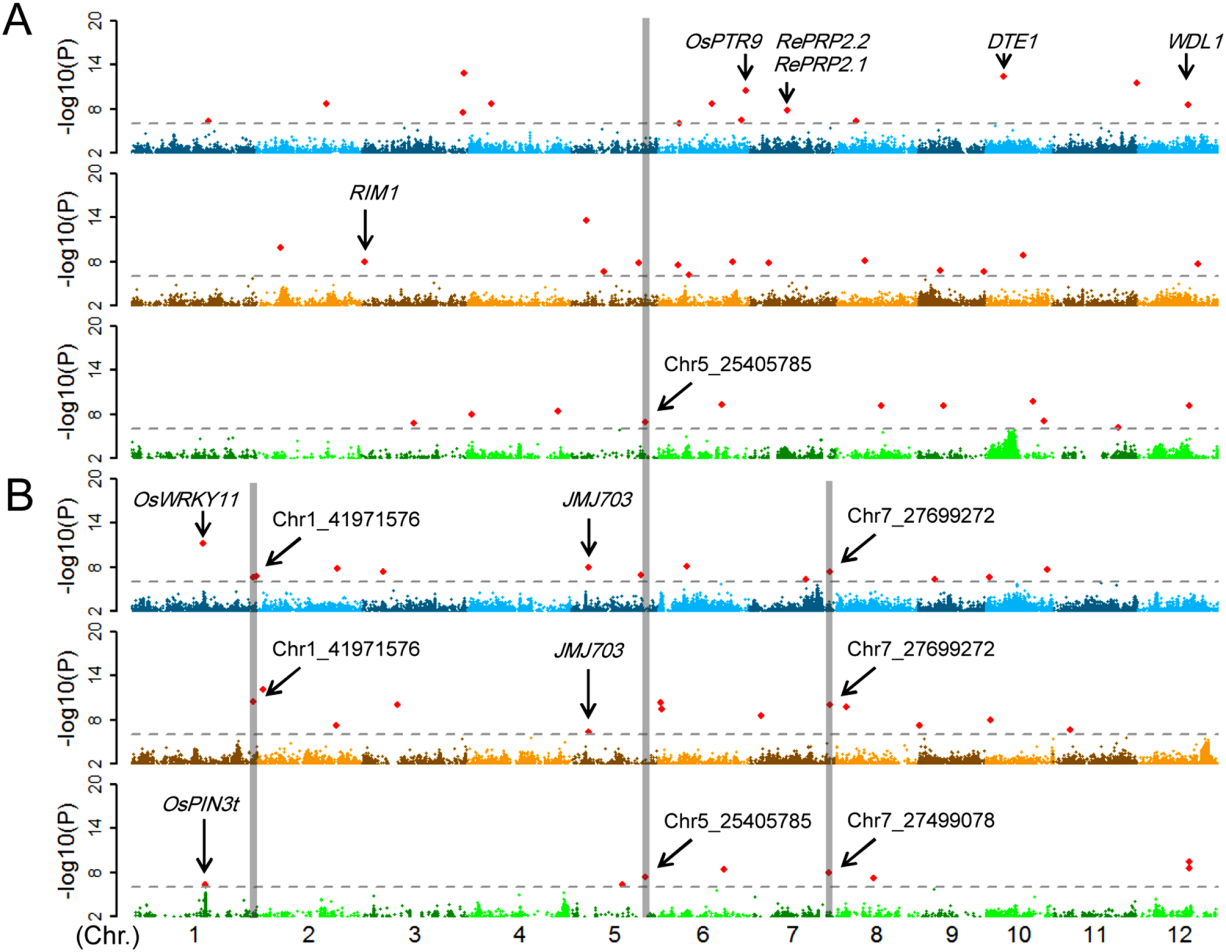
identification of root weight and shoot weight QTLs by GWAS. Manhattan plots in full population (blue), *indica* (yellow) and *japonica* (green) for (**A**) root weight and (**B**) shoot weight. Nine known genes around corresponding association signals were marked, together with one comment signal (Chr5_25405785) and two signal-enriched clusters for shoot weight (Chr1_41971576, Chr7_27699272 and Chr7_27499078).

Rice biomass is a complex trait that can be dissected into root, shoot and R/S. The three traits can reflect relative biomass allocation between roots and shoot. A total of 39 and 32 association signals for root weight and shoot weight were directly detected along with 25 R/S association signals in the full population and two subpopulations (Fig. 3, Table S3). Among these two new shoot weight association signals (Chr1_41971576 and Chr7_27699272) and one known gene *JMJ703* around Chr5_6020893 were identified in the full population and subpopulations (Fig. 3). Previous study indicated that *JMJ703* was indispensable for rice development, and its mutants displayed pleiotropic phenotypes, the most obvious of which was dwarfism^47^. Importantly, a new common association signal was detected for root weight and shoot weight at Chr5_25405785 in *japonica*, which involved in regulation of coordinated development of roots and shoots (Fig. 3).

Long distance pleiotropic signals play important roles in mediation of shoot–root communication^48^. The local LD was examined around the association signal (Chr5_25405785) to explore causal gene for root weight and shoot weight. We estimated a candidate region from 25.2 to 25.5 Mb on chromosome 5 by using pairwise LD correlations (*R*^2^ ≥ 0.6) (Fig. S11). There were 15 annotated genes within the candidate region, and 13 of which showed stable expression in root and shoot of different rice accessions (Table S4). Of the 13 genes, Loc_Os05g43450 gene (also predicted as Os05g0509900 and referred to hereafter as *OsTFIIF2-1*) is the most likely candidate gene for root weight and shoot weight, since it encodes a subunit of RNA polymerase II which is a prerequisite for normal development in plants^49^.

**Figure 4:**
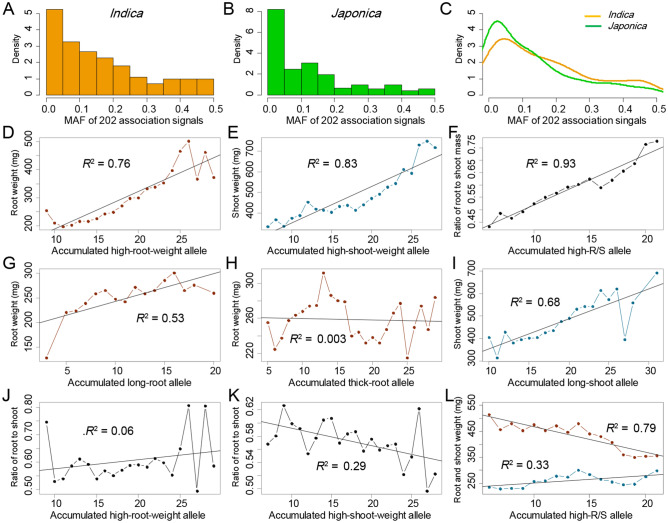
Dissection of genetic basis underlying rice biomass. Characterization of allele frequencies at 202 association signals in (**A**) *indica* and (**B**) *japonica*, and (**C**) comparison of them between both rice subpopulations. Plots of the (**D**) root weight, (**E**) shoot weight and (**F**) ratio of root-to-shoot mass against the accumulation of their corresponding to superior alleles. Plots of the (**G**,**H**) root weight and (**I**) shoot weight against the accumulation of their sub-traits superior alleles. Plots of the ratio of root-to-shoot mass against the accumulation of (**J**) root weight and (**K**) shoot weight superior alleles. (**L**) Plot of ratio of root-to-shoot mass against root weight and shoot weight.

There were different MAF among 39, 32 and 25 association signals for root weight, shoot weight and R/S, respectively (Table S5). Obvious differences in allele frequency were also observed between both subpopulations in most association signals (Fig. 4A–C, Table S5). Importantly, 3 *indica*-specific and 2 *japonica*-specific alleles respectively were detected out of the 39 root weight association signals (Table S5). Meanwhile, *indica*-specific allele variations were identified at 2 out of 32 shoot weight association signals, and *japonica*-specific allele variation was explored at 1 out of 25 R/S association signals. Advanced differentiation at these loci between both subpopulations can explain why there is no common biomass accumulation association signal between *indica* and *japonica*. In addition, there were large variations in a number of accumulated superior alleles of all association signals corresponding to each of the three traits in the rice panel (Fig. 4D–F). We further observed that the root weight, shoot weight and R/S increased with the accumulation of high-root-weight, high-shoot-weight and high-R/S alleles of corresponding association signals with *R*^2^ at 0.76, 0.83 and 0.93, respectively (Fig. 4D–F). These suggest that root weight, shoot weight and R/S are controlled mainly by additive effects of causal genes. Comprehensively, we propose that high-biomass breeding could be achieved through pyramiding high-root-weight and high-shoot-weight alleles distributing within different rice subpopulations.

Root length and thickness are important sub-traits of root weight. While shoot length is a main sub-trait of shoot weight. There were totally 26, 42 and 39 association signals for root length, root thickness and shoot length, respectively. The linear relations were also observed between these traits and accumulation of superior alleles of their respective association signals with *R*^2^ at 0.98, 0.77 and 0.94, respectively. (Fig. S12). Importantly, we also observed that root weight increased with the accumulation of long-root alleles of corresponding association signals, while shoot weight increased with the accumulation of long-shoot alleles of corresponding association signals with *R*^2^ at 0.53 and 0.68, respectively (Fig. 4G,I). However, there was no clear increase of root weight with the accumulation of thick-root alleles of corresponding association signals (Fig. 4H). These results showed that longitudinal growth (root and shoot length) was one key factor for biomass accumulation of root and shoot, and pyramid of long-root and long-shoot alleles can also contribute to high-biomass breeding in rice.

As a complex trait, R/S was involved in root weight and shoot weight. However, no obvious linear relationships were detected between R/S and accumulation of high-root-weight alleles of root weight association signals, and between R/S and accumulation of high-shoot-weight alleles of shoot weight association signals in rice (Fig. 4J,K). On the other hand, with the accumulation of high-R/S alleles of R/S association signals, the shoot weight gradually decreased while the root weight remained unchanged (Fig. 4L). To explain this phenomenon, we investigated the correlation between numbers of accumulated high-root-weight alleles and accumulated high-shoot-weight alleles from their corresponding association signal. Moderate correlations exist between them (Pearson's correlation coefficient at 0.544, 0.355 and 0.618 in full population, *indica* and *japonica*) which indicated that there was a similar trend for accumulation of high-root-weight alleles and accumulated high-shoot-weight alleles from their corresponding association signal in rice germplasm. These results suggested that there could be a delicate balance of high-root-weight alleles and high-shoot-weight allele, which was determined by pleiotropy, LD, selection and maybe other factors.

### Genetic network of root and shoot loci association

We observed association signals for six biomass traits accumulation unevenly distributed across the rice genome (Fig. 2). In this study, we defined an enriched cluster including two significant SNP clusters within strong LD 340 kb apart. Further investigation indicated that 89 of 202 association signals were clustered within 37 enriched clusters, except for one common association signal (Chr5_25405785) for root and shoot weight in *japonica* and two association signal (Chr1_41971596 and Chr7_27699272) for shoot weight in *indica* and full population (Table S6). Among these enriched clusters, there were three enriched clusters related at least two shoot traits (containing seven association signals). On the other hand, ten enriched clusters related at least two root traits (containing 21 association signals) and 11 enriched clusters related at least one root trait and one shoot trait (containing 31 association signals), along with five enriched clusters for either one root or shoot trait (containing ten association signals) (Table S6). Additionally, five enriched clusters with ten signals were associated with R/S and one sub-trait, and one enriched cluster with two signals was associated with R/S (Table S6). A large number of enriched clusters related to root and shoot traits confirmed that these causal genes are not randomly distributed, but are more likely to aggregate into strong LD functional modules.

**Figure 5:**
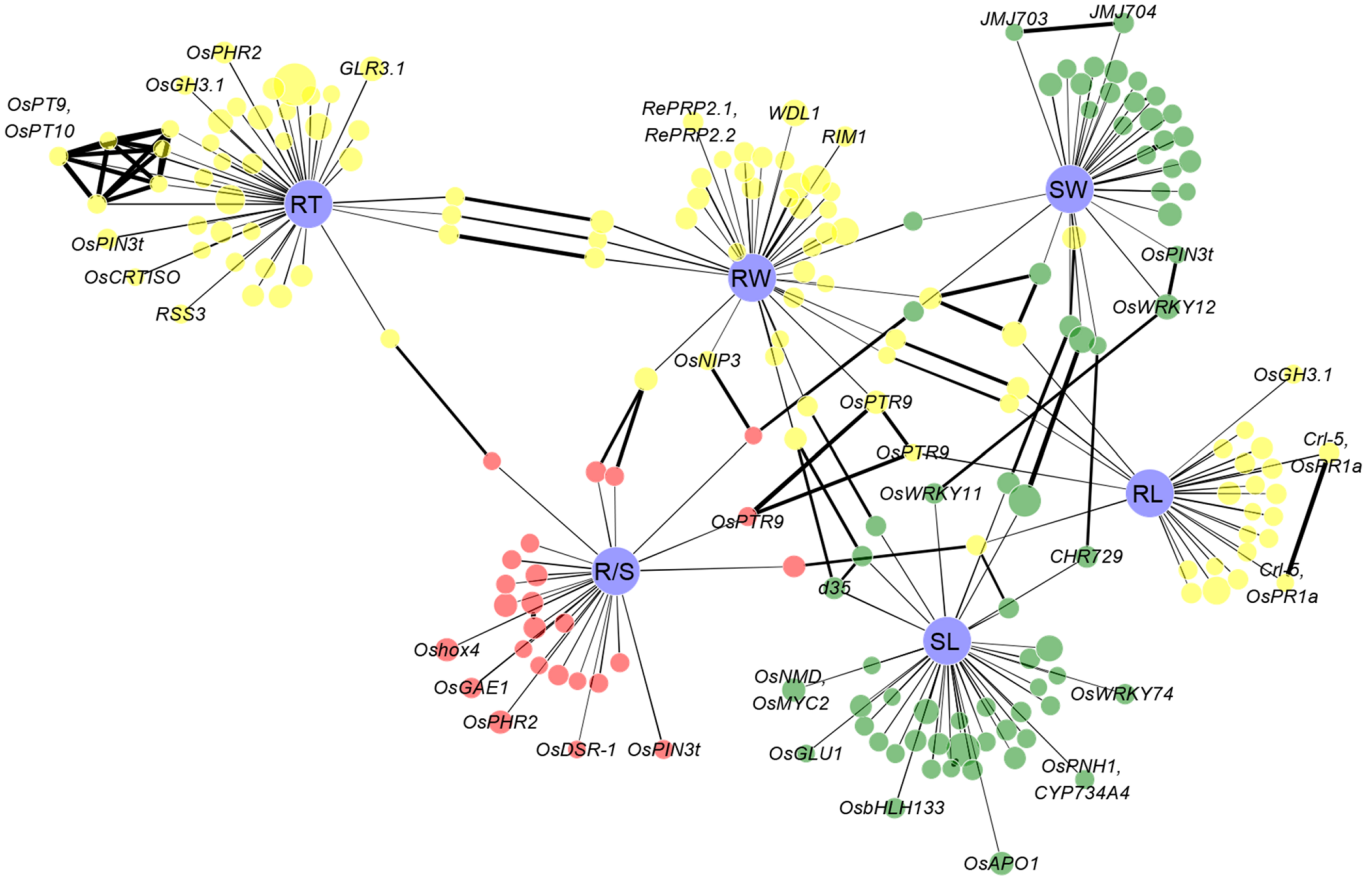
Genome-wide association network for six rice biomass traits. Six traits were represented by violet nodes, and association signals for three root traits (root weight (RW), root length (RL) and root thickness (RT)), two shoot traits (shoot weight (SW) and shoot length (SL)) and ratio of root-to-shoot mass (R/S) were presented by yellow, green and red nodes, respectively. The size of nodes showed the − log(*p*) value of corresponding association signals (Table 1). Link between trait and association signal indicated their association. The links between association signals for different biomass traits indicated strong LD with *R*^2^ > 0.2, and the thickness of link showed LD value (Table 1).

We analyzed the association network of all six traits in rice to further confirm the roles of LD and pleiotropy in correlations among different traits related to roots and shoots biomass accumulation (Fig. 5). There were 24 pairs of association signals for different traits among 202 association signals and each pair has obvious LD with *R*^2^ range from 0.2 to 0.9 (Table 1). Interestingly, five known genes were around the 24 pairs of association signals, including *OsWRKY11*, *d35*, *OsPTR9*, *CHR729*, and *OsNIP3* (Table 1). Of these, *OsWRKY11* which encodes a WRKY transcription factor controlling plant height and flowering was identified around association signals Chr1_34783550 for shoot length and Chr1_24803735 for shoot weight^15^. Similarly, *OsPTR9* was around Chr6_30045542 for root weight, Chr6_30047965 for R/S and Chr6_30077572 for root length. The elevated expression of *OsPTR9* in transgenic rice plants resulted in enhanced ammonium uptake, promotion of lateral root formation and increased grain yield^50^. Additionally, *d35* was around Chr6_21936682 for shoot length with moderate LD to Chr6_21687213 for root weight at *R*^2^ = 0.28, which regulated plant height in rice^11^. *CHR729* was around Chr7_18407253 for shoot length with moderate LD to Chr7_19348504 for shoot weight at *R*^2^ = 0.37, and its corresponding mutant line exhibited late seed germination, low germination rate, dwarfism, low tiller number, root growth inhibition^51, 52^. *OsNIP3* was around Chr10_19886882 for root weight with moderate LD to Chr10_20880089 for R/S, which is expressed mainly in exodermal cells and steles in roots, and critical for vegetative growth and reproductive development in rice grown under boron-deficient conditions^53, 54^. These results confirmed that the root and shoot biomass was co-regulated by these QTLs, pleiotropy and LD play important roles in synergistic development of roots and seedlings in rice.

**Table 1:** Twenty-four pairs of association signals for different rice biomass traits with strong LD.

Signal_A			Signal_B			*R*^2^
Position	Trait	Known gene around signal_A	Position	Trait	Known gene around signal_B	
Chr1_22317253	Root thickness	–	Chr1_23104043	Ratio of root to shoot	–	0.37
Chr1_24783550	Shoot length	*OsWRKY11*	Chr1_24803735	Shoot weight	*OsWRKY11*	0.27
Chr2_2181734	Shoot length	–	Chr2_2190465	Shoot weight	–	0.90
Chr2_7382634	Ratio of root to shoot	–	Chr2_7969845	Root weight	–	0.48
Chr2_7969845	Root weight	–	Chr2_8392444	Ratio of root to shoot	–	0.52
Chr3_17596162	Root thickness	–	Chr3_18010651	Root weight	–	0.23
Chr6_1090424	Shoot weight	–	Chr6_1118367	Shoot length	–	0.48
Chr6_21574106	Shoot length	–	Chr6_21687213	Root weight	–	0.41
Chr6_21687213	Root weight	–	Chr6_21936682	Shoot length	*d35*	0.28
Chr6_30045542	Root weight	*OsPTR9*	Chr6_30047965	Ratio of root to shoot	*OsPTR9*	0.86
Chr6_30045542	Root weight	*OsPTR9*	Chr6_30077572	Root length	*OsPTR9*	0.63
Chr6_30047965	Ratio of root to shoot	*OsPTR9*	Chr6_30077572	Root length	*OsPTR9*	0.57
Chr7_6836133	Root thickness	–	Chr7_6849331	Root weight	–	0.82
Chr7_18407253	Shoot length	*CHR729*	Chr7_19348504	Shoot weight	–	0.37
Chr8_6201957	Root length	–	Chr8_6966865	Root weight	–	0.29
Chr10_13106609	Root length	–	Chr10_13212543	Ratio of root to shoot	–	0.27
Chr10_16372108	Root thickness	–	Chr10_16449959	Root weight	–	0.49
Chr10_19886882	Root weight	*OsNIP3*	Chr10_20880089	Ratio of root to shoot	–	0.38
Chr10_20880089	Ratio of root to shoot	–	Chr10_21256365	Shoot weight	–	0.31
Chr12_17215000	Root length	–	Chr12_17311797	Root weight	–	0.52
Chr12_17845083	Root weight	–	Chr12_17848236	Shoot weight	–	0.72
Chr12_17845083	Root weight	–	Chr12_17869886	Root length	–	0.70
Chr12_17848236	Shoot weight	–	Chr12_17869886	Root length	–	0.57
Chr12_20594911	Shoot length	–	Chr12_20854338	Root weight	–	0.31

### Close intragenic linkage of elite natural alleles in known pleiotropic gene *OsPTR9*

Exploration of elite alleles for these known genes around corresponding signal is conducive to its better utilization in rice biomass breeding. In this study, we focused on non-synonymous SNPs, due to polymorphisms differentiating causal protein-coding differences are most likely to be important functional loci associated with agronomic traits^55, 56^. A total of 623 non-synonymous SNPs were detected within the 30 known genes of the association panel. Among them, 13 non-synonymous SNPs (MAF > 0.05) within five known genes showed significant association with corresponding traits under general linear model (*p* < 0.01) (Table 2).

**Table 2:** Summary of 13 possible functional SNPs within five known genes.

Trait	Signal under FarmCPU	Known gene	Non synonymous SNP in known gene	MAF	Alleles	Elite allele	− log(*P*) under GLM		
							Full population	*Japonica*	*Indica*
Root weight	Chr6_30045542	*OsPTR9*	Chr6_29839744	0.23	A/T	T	7.2	3.36	–
			Chr6_29840728	0.24	A/C	C	6.67	4.19	–
			Chr6_29840758	0.24	T/C	C	6.22	3.92	–
R/S	Chr6_30047965	*OsPTR9*	Chr6_29839744	0.23	A/T	T	8.82	4.11	–
			Chr6_29840728	0.24	A/C	C	7.69	3.83	–
			Chr6_29840758	0.24	T/C	C	7.36	3.74	–
Root length	Chr6_30077572	*OsPTR9*	Chr6_29839102	0.13	A/G	G	2.23	–	2.86
	Chr7_1278547, Chr7_1244488	*Crl-5, OsPR1a*	Chr7_1303391	0.44	C/G	C	2.09	–	2.41
Root thickness	Chr4_29374382	*GLR3.1*	Chr4_29566027	0.38	A/C	A	2.63	–	2.2
			Chr4_29567859	0.39	C/T	T	2.86	–	2.07
			Chr4_29567998	0.38	A/G	G	3.02	–	2.23
			Chr4_29569521	0.4	C/T	C	2.11	–	2.58
Shoot length	Chr1_24783550	*OsWRKY11*	Chr1_25011608	0.47	A/G	A	5.75	–	3.47
	Chr6_21936682	*d35*	Chr6_22074153	0.36	G/C	G	6.54	3.28	–
			Chr6_22074288	0.3	A/G	A	4.61	2.57	–
			Chr6_22076053	0.36	A/G	G	6.5	2.91	–

**Figure 6:**
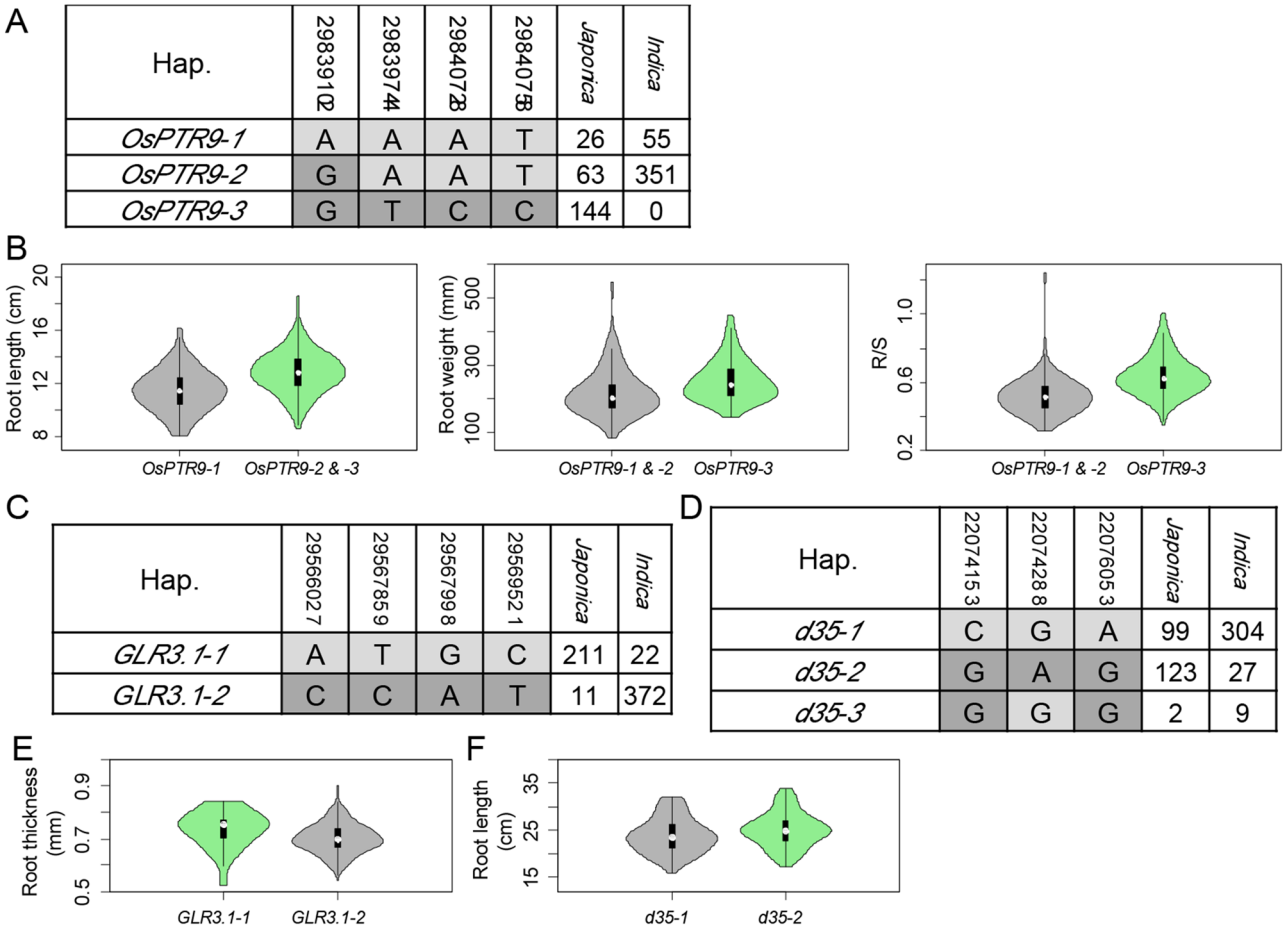
Example of discrimination between intragenic linkage and true pleiotropy by haplotype analysis of known rice biomass genes *OsPTR9*, *GLR3.1* and *D35*. Gene structure of (**A**) *OsPTR9*, (**C**) *GLR3.1* and (**D**) *d35*, and comparisons of different traits among (**B**) *OsPTR9*, (**E**) *GLR3.1* and (**F**) *d35* genotypes.

Four non-synonymous SNPs were identified within *OsPTR9* gene, which are associated with root weight, R/S and root length in this study (Table S3). The results agree to that it plays an important role in controlling nitrogen uptake and lateral root development in rice^50, 57^. Interestingly, three non-synonymous SNPs out of four (Chr6_29839744, Chr6_29840728 and Chr6_29840758) showed association with root weight and R/S with extremely low *p* value, and one non-synonymous SNP (Chr6_29839102) was associated with root length (Table 2). Allele T at Chr6_29839744, allele C at Chr6_29840728 and allele C at Chr6_29840758 showed more root weight and higher R/S While allele G at Chr6_29839102 showed longer root length (Table 2). The results suggested that pleiotropic phenotypes of *OsPTR9* could be determined by several non-synonymous SNPs, and each non-synonymous SNP was responsible for a different trait. Based on the four non-synonymous SNPs, we further identified three main haplotypes *OsPTR9-1* to *OsPTR9-3* (Fig. 6A). Among them, *OsPTR9-3* was *japonica*-specific predominant haplotype, and extremely significant differences of root weight and R/S were detected between *OsPTR9-3* and other two haplotypes in *japonica* with *p* value less than 1.0 × 10^−4^ and 1.0 × 10^−6^, respectively (Fig. 6B)*.* Meanwhile, the root length of *OsPTR9-1* was shorter than that of other two haplotypes in *indica* with *p* value less than 1.0 × 10^−5^ (Fig. 6B). Meanwhile, close intragenic linkage was detected among three possible functional sites of SNPs within *OsPTR9* of *japonica* (Chr6_29839744, Chr6_29840728 and Chr6_29840758) and were responsible for root weight and R/S. A forth site of SNPs (Chr6_29839102) was for root length. Taken together, this study supported that *OsPTR9* was a pleiotropy gene for root weight, R/S and root length, and there were close intragenic linkage among the three possible functional SNPs underlying root weight.

Additionally, four non-synonymous SNPs (Chr4_29566027, Chr4_29567859, Chr4_29567998 and Chr4_29569521) were identified to be associated with root thickness within *GLR3.1* gene (Fig. 6C and Table 2), which was disrupted by T-DNA insertion resulting in shorter root^58^. Based on the four non-synonymous SNPs, two main haplotypes were identified in cultivated rice, and significant difference of root thickness was detected between *GLR3.1-1* and *GLR3.1-2* in *indica* with *p* value less than 0.05 (Fig. 6E). Close intragenic linkage was also detected among four possible functional sites of SNPs within *GLR3.1-2*. Meanwhile, three non-synonymous SNPs (Chr6_22074153, Chr6_22074288 and Chr6_22076053) were identified to associate with shoot length within *d35*, of which *d35*^*Tan-Ginbozu*^ mutant was only 60–70% the height for the parental cultivar throughout the vegetative development^11^. Based on the three non-synonymous SNPs, two main haplotypes were identified in cultivated rice (Fig. 6D and Table 2), and *japonica* accessions carrying *d35-2* showed long shoot length than accessions carrying *d35-1* (Fig. 6F). In conclusion, close intragenic linkage is a universal phenomenon in rice, and close intragenic linkage of QTNs for different traits play an important role in gene pleiotropy and trait correlation. The intragenic linkage enables fine-tuning on synergistic development of roots and seedlings at the sequence polymorphism level.

### The *OsPTR9-3* allele is conservative and unique to temperate *japonica* originated from *japonica*-like wild rice

**Figure 7:**
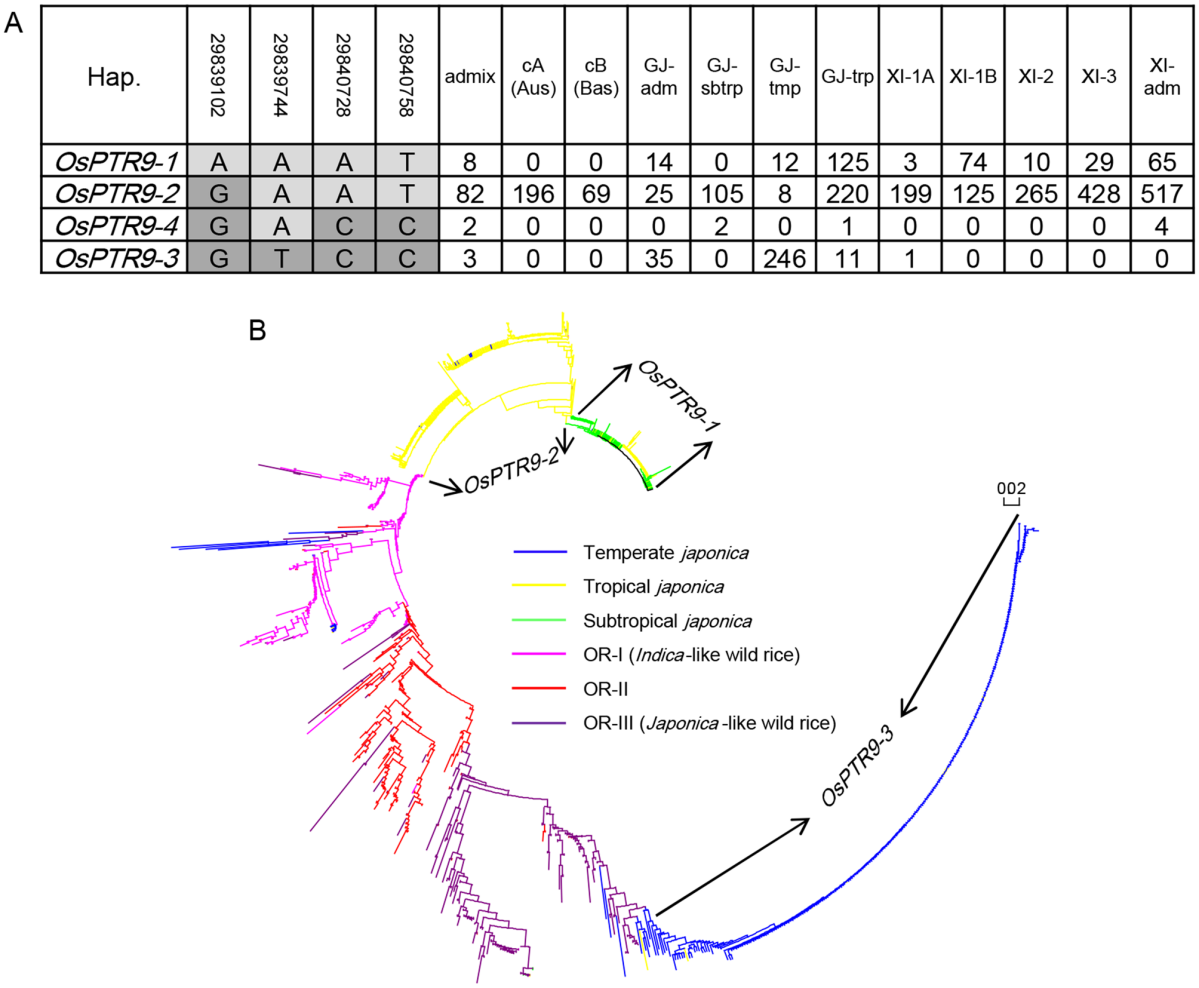
Domestication process of *OsPTR9*. (**A**) Haplotype analysis and (**B**) phylogenetic relationship analysis using 3024 cultivated and 446 wild rice accessions. According to previous studies, XI-1A is *indica* subpopulation 1A, XI-1B is *indica* subpopulation 1B, XI-2 is *indica* subpopulation 2, XI-3 is i*ndica* subpopulation 3, XI-adm is *indica* admixed types between two or more XI subpopulations, GJ-adm is *japonica* admixed types between two or more GJ subpopulations, GJ-trp is japonica tropical subpopulation, GJ-sbtrp is japonica subtropical subpopulation, GJ-tmp japonica temperate subpopulation, cA or Aus is centrum-Aus population, cB or Bas is centrum-Basmati population and admix is admixed between any two or more of the XI, GJ, cA (Aus), cB (Bas) populations.

A total of 3,024 cultivated and 446 wild rice accessions were used to further determine how the rice accessions carrying *OsPTR9-3* allele are distributed and formed. There were 12 main subpopulation in cultivated rice panel (Fig. 7A), and 3 main subpopulation in wild rice panel (Fig. 7B) according to previous studies^16, 17^. Four haplotypes were identified within the large cultivated rice panel based on the four non-synonymous SNPs, including one rare *OsPTR9-4* which only exists in nine accessions (Fig. 7A). The results further confirmed close intragenic linkage within the pleiotropy gene *OsPTR9*, especially within the three candidate functional loci for root weight Chr4_29567859, Chr4_29567998 and Chr4_29569521. Of 296 rice accessions carrying *OsPTR9-3*, 148 were temperate *japonicas* from East Asia (Japan, South Korea, North Korea, China) (Fig. 7A and Table S7). Meanwhile, one definite and 13 possible carriers of *OsPTR9-3* allele were identified in *japonica*-like wild rice, which were mainly distributed in the middle area of the Pearl River in southern China (Table S8).

A phylogenetic analysis and selection were performed to further determine the domestication history of *OsPTR9* in *japonica*, using 34 SNPs of temperate *japonica*, tropical *japonica*, subtropical *japonica* and wild rice. Distinct differentiations were detected between *japonica* accessions carrying *OsPTR9-3* and other haplotypes, based on phylogenetic tree from SNPs in *OsPTR9-3* (Fig. 7B). Temperate *japonica* accessions carried *OsPTR9-3* were close to *japonica*-like wild rice, but tropical and subtropical *japonica* accessions carried *OsPTR9-1* and *-2* were close to *indica*-like wild rice (Fig. 7B). The results further confirmed the origin of *OsPTR9-3* allele from *japonica*-like wild rice of Guangxi, China, but *OsPTR9-1* and *-2*, which was prevalent in tropical and subtropical *japonica* and *indica*, could be derived from *indica*-like wild rice of South Asia. Selective signal scans of *OsPTR9* were performed within distinct subpopulations. Lowest nucleotide diversity (0.0004) and Tajima’s *D* (− 2.56) were identified in temperate *japonica* accessions carrying *OsPTR9-3* haplotype (Table 3). These findings suggested the strong directional selection across the gene in temperate *japonica* could be one cause for its conservativeness and uniqueness in temperate *japonica* rice.

**Table 3:** Nucleotide diversity and Tajima’s *D* Test of *OsPTR9.*

Taxon	π	Tajima's *D*
Cultivated rice	0.0043661	− 0.100662
*Indica* accessions carrying *OsPTR9-1*	0.0007372	− 1.543521
*Indica* accessions carrying *OsPTR9-2*	0.0014426	− 1.592862
*Indica*	0.0020698	− 1.448557
*Japonica* accessions carrying *OsPTR9-1*	0.0009423	− 1.302912
*Japonica* accessions caryring *OsPTR9-2*	0.0024237	0.0752065
*Japonica* accessions carrying *OsPTR9-3*	0.0003837	− 2.560482
*Japonica*	0.0062616	1.4971241
Wild rice	0.002484	− 0.403569

## Discussion

### Regulation of synergistic roots and shoots biomass accumulation in rice

The complex traits (biomass) are determined by multiple sub-traits (root weight, shoot weight and R/S) and each sub-trait is further determined by a second-order trait (root length, root thickness and shoot length) through the utilization of two main genetic regulation approaches. One is the top-to-bottom regulation model which determined the highest priority biomass regulation pathway of root weight, shoot weight and R/S, followed by lower priority pathway for root weight, shoot weight. Based on the present study, we also proposed a bottom-to-top regulation model which determined the lowest-order traits (root length, root thickness and shoot length) its own regulation loci and competition among different traits, as well as the multiple validity and LD of control loci and also ensured the coordinated development of each trait and the accumulation of total biomass. Integrated intracellular and intercellular signaling networks through roots and shoots are considered as top-to-bottom regulation model for plant growth^27–29^. For example, the endogenous level of gibberellin (GA) and/or the GA sensitivity of shoots and roots plays a role in determining the shoot-to-root ratio of the plant^59^. Cytokinin and auxin are involved in meristem development and maintenance of amount for both roots and shoots^60–62^. However, the top-to-bottom regulation model is inadequate to explain the synergistic biomass accumulation of roots and shoots in rice to full extent, due to large variations in a lowest-order single trait (root length, root thickness or shoot length) which are sufficient to affect final biomass in rice^9, 14, 63, 64^.

In this study, the highest correlations were detected between root weight and shoot weight in all three populations (Figs. 1D, S4). Meanwhile, a total of 39 and 32 association signals for root weight and shoot weight were directly detected with 25 R/S association signals (Fig. 3), and fewer of them showed association with each other or association with their sub-traits. Further considering the highest additive effects between traits (root weight, shoot weight and R/S) and the accumulation of their corresponding excellent alleles (Fig. 4), we conclude that top-to-bottom regulation model could play an important regulatory role in synergistic biomass accumulation of roots and shoots. However, we also detected moderate correlations between traits and their corresponding sub-traits, and moderate additive effects between traits and the accumulation of excellent alleles corresponding to its sub-traits, suggesting a bottom-to-top regulation model was possibly also involved in synergistic biomass accumulation of roots and shoots. The study of these two regulatory models points out a promising direction for a better understanding of the genetic basis and network underlying synergistic biomass accumulation of roots and shoots in rice. Nevertheless, it remains a challenge to understand which model could be predominant and how different one model controls rice biomass at the molecular level and how environment factors fit in a particular model.

### The role of pleiotropy and LD in correlation among traits related to rice biomass accumulation

The consequences of trait correlations are the most important issues to be considered in breeding, such as the negative correlation between grain number per panicle and panicle number, and between grain number per panicle and kilo-grain weight^25^, positive correlation between heading date and grain yield per plant, and between heading date and grain number per panicle^25, 65^ and positive correlation between root weight and shoot weight in this study. Pleiotropy and LD are two considerable factors responsible for trait correlations, and it is hard to distinguish them in a short physical interval. There are two types of genes with genic pleiotropy: one is a gene with a single function, but involved in multiple biological processes, and another is a gene with multiple functions that contribute to different traits^30^. At present, it is still more difficult to utilize effectively the pleiotropic gene, because it usually cause the un-decomposable correlation between favorable traits and disadvantageous traits^25^. By comparison, physical linkage and association of non-linked genes can be cut off by modern biotechnology to achieve effective utilization of favorable genotypes.

It is feasible to solve gene pleiotropy at the level of sequence polymorphisms, because a gene with single function is most likely due to a pleiotropic QTN, whereas intragenic linkage of QTNs seems more likely the underlying cause of genic pleiotropy in the case of a gene with multiple functions^30^. In fact, some studies have shown that gene pleiotropy is caused by the different allele combinations of intragenic QTNs, such as *SD1* for plant height and internode growth of stems to keep above the water in rice^14, 66^, and *Dwarf8* for plant height and flowering time in maize^67^. In this study, a total of 37 enrich genome region related to at least two traits, and one common association signal for root and shoot weight were identified (Table S6), which confirmed important role of pleiotropy and LD in synergistic biomass accumulation of roots and shoots in rice. Further analysis of known pleiotropy gene *OsPTR9*, provided a successful example of discrimination between intragenic linkage and true pleiotropy with impact on correlations among biomass traits, due to close linkage among the four non-synonymous SNP (Chr4_29566027 associated with root length and Chr4_29567859, Chr4_29567998 and Chr4_29569521 associated with root weight) within *OsPTR9*. Importantly, deep analysis of the role of pleiotropy and LD in correlation among traits provides guidance for fine tuning of complex traits in crop improvement by careful manipulation of cloned QTL genes at the sequence level using various molecular technologies.

## Supplementary Information

Supplementary Figures.

Supplementary Table 1.

Supplementary Table 2.

Supplementary Table 3.

Supplementary Table 4.

Supplementary Table 5.

Supplementary Table 6.

Supplementary Table 7.

Supplementary Table 8.

## Data Availability

Genome sequencing data of 666 rice accessions are obtained from the 3KRGP^16^, and all phenotypic data are at Supplementary Table S1.
