# Supplementary material for: Genetic basis and network underlying synergistic roots and shoots biomass accumulation revealed by genome-wide association studies in rice
Source: Sci Rep. 2021 Jul 2;11:13769. doi: 10.1038/s41598-021-93170-3 (PMC8253791; doi:10.1038/s41598-021-93170-3)
Supplement: Supplementary file 1 — Supplementary Figures. [file 41598_2021_93170_MOESM1_ESM.docx]

**Supplementary Figures**

**Genetic basis and network underlying synergistic roots and shoots biomass accumulation revealed by genome-wide association studies in rice**

Yan Zhao^1,2*^, Zhigang Yin^1*^, Xueqiang wang^1*^, Conghui Jiang^1*^, Muhammad Mahran Aslam^1^, Fenghua Gao^1^, Yinghua Pan^3^, Jianyin Xie^1^, Xiaoyang Zhu^1^, Luhao Dong^2^, Yanhe Liu^2^, Hongliang Zhang^1^, Jinjie Li^1^, Zichao Li^1#^

^1^ Key Laboratory of Crop Heterosis and Utilization of the Ministry of Education, and Beijing Key Laboratory of Crop Genetic Improvement, China Agricultural University, Beijing 100193, China.

^2^ State Key Laboratory of Crop Biology, Shandong Key Laboratory of Crop Biology, College of Agronomy, Shandong Agricultural University, Tai'an, Shandong, 271018, PR China.

^3^ Guangxi Key Laboratory of Ｒice Genetics and Breeding, Rice Research Institute of Guangxi Academy of Agricultural Sciences, Nanning, Guangxi, 530007, China.

^*^ These authors contributed equally to this work.

^#^ Correspondence should be addressed to Zichao Li (Email: [lizichao@cau.edu.cn](mailto:lizichao@cau.edu.cn); Tel: +86 010 62731414).

**Fig. S1 Population structure of association panel.**

**(A)** Genetic structure of the panel at *K* = 2 using the software Admixture. **(B)** A neighbor-joining tree of the panel, green and yellow show *japonica* and *indica*, respectively. Black dot indicates one African cultivated rice accession (*Oryza glaberrima* L.). **(C and D)** Principle components analysis reveals that the first 3 principle components explain 29% of the genetic variation within the panel.

**Fig. S2 Histograms of the six traits for biomass accumulation in root and shoot in full population (blue), *indica* (yellow) and *japonica* (green) subpopulations.**

**Fig. S3 Comparison of the six traits for biomass accumulation in root and shoot between *indica* (yellow) and *japonica* (green) subpopulations.**

**Fig. S4 A heatmap depicting Pearson’s correlation coefficients between phenotype means in full population for the six traits.**

**Fig. S5 Quantile-quantile (QQ) plots of FarmCPU for six traits in full population.**

**Fig. S6 Manhattan plots for ratio of root-to-shoot mass, shoot length, root length and root thickness (in accordance with the order of top-to-bottom model) in the full population.**

**Fig. S7 QQ plots of FarmCPU for the six traits in the *indica* subpopulation.**

**Fig. S8 QQ plots of FarmCPU for the six traits in the *japonica* subpopulation.**

**Fig. S9 Manhattan plots for ratio of root-to-shoot, shoot length, root length and root thickness (in accordance with the order in top-to-bottom model) in the *indica* subpopulation.**

**Fig. S10 Manhattan plots for ratio of root-to-shoot, shoot length, root length and root thickness (in accordance with the order in top-to-bottom model) in the *japonica* subpopulation.*P***

**Fig. S11 A heatmap depicting linkage disequilibrium (LD) around the association signal (Chr5_25405785) for root weight and shoot weight.**

**Fig. S12 Plots of the root length (left), root thickness (middle) and shoot length (right) against the accumulation of their corresponding superior alleles.**
