# Supplementary material for: Genetic basis and network underlying synergistic roots and shoots biomass accumulation revealed by genome-wide association studies in rice
Source: Sci Rep. 2021 Jul 2;11:13769. doi: 10.1038/s41598-021-93170-3 (PMC8253791; doi:10.1038/s41598-021-93170-3)
Supplement: Supplementary file 3 — Supplementary Table 2. [file 41598_2021_93170_MOESM3_ESM.docx]

**Supplementary Table 2. Differencesof root and shoot traits between the *indica* and *japonica* subgroups.**

| Subgroup | Root weight | Shoot weight | Root length | Root thickness | Shoot length | R/S |
| --- | --- | --- | --- | --- | --- | --- |
| *Japonica* (252) | 242.8±67.0 mg | 401.1±102.0 mg | 12.83±1.49 cm | 0.73±0.07 mm | 24.77±3.85 cm | 0.59±0.13 |
| *Indica* (414) | 276.5±71.7 mg | 469.4±106.8 mg | 12.61±1.64 cm | 0.70±0.06 mm | 25.31±3.80 cm | 0.57±0.10 |
| T-test (Two-tail) | 2.66E-09 | 2.02E-15 | 0.082 | 2.78E-08 | 0.075 | 0.045 |
